# Determinants of asthma in Ethiopia: age and sex matched case control study with special reference to household fuel exposure and housing characteristics

**DOI:** 10.1186/s40733-021-00080-2

**Published:** 2021-11-25

**Authors:** Yonas Abebe, Ahmed Ali, Abera Kumie, Tewodros Haile, Mulugeta Tamire, Adamu Addissie

**Affiliations:** 1 Department of Preventive Medicine, School of Public Health, College of Health Sciences, Addis Ababa University, Addis Ababa, Ethiopia; 2 Department of Internal Medicine, School of Medicine, College of Health Sciences, Addis Ababa University, Addis Ababa, Ethiopia

**Keywords:** Household fuel exposure, Biomass fuel, Housing characteristic, Matched case control, Asthma, Ethiopia

## Abstract

**Background:**

Asthma is a chronic inflammatory disorder characterized by airway obstruction and hyper-responsiveness. Studies suggest that household fuel exposure and housing characteristics are associated with air way related allergy. But there remains to be a considerable uncertainty about whether that reflects an association with asthma. This study endeavored to bridge the gap by identifying factors associated with asthma, with special reference to household fuel exposure and housing characteristics in selected public hospitals in Addis Ababa, Ethiopia.

**Methods:**

We conducted a hospital-based matched case-control study. A total of 483 study participants were selected from two Ethiopian referral hospitals using a sequential sampling technique, with 161 cases and 322 controls. Standard questionnaire from the European Community Respiratory Health Survey II (ECRHS II) and the American Thoracic Society Division of Lung Disease (ATS-DLD-78) were used to collect household related data. Conditional logistic regression model was applied to identify the determinants of asthma. Both crude and adjusted odds ratios with 95% confidence interval (CI) were used to identify predictors of asthma.

**Results:**

The response rate for both cases and controls was 99.17%. The odds of developing asthma was about four times higher among those who used agricultural residues for cooking (AOR: 3.81, 95% CI: 1.05, 13.79)., about five times higher among those who used wood for cooking (AOR: 4.95, 95% CI: 2.1, 11.69), nearly five times higher among those who had family history of asthma (AOR: 4.72, 95% CI: 1.54, 14.45), just over six times higher among those who smoke tobacco (AOR: 6.16, 95% CI: 1.31, 29.09) and over ten times higher among those who do not practice door opening, while cooking (AOR: 10.25, 95% CI: 3.97, 26.49).

**Conclusion:**

Family history of asthma, tobacco smoking, use of solid fuels including, woods and agricultural residues were associated with development of asthma. To reduce the risk of asthma, people should practice door opening, while cooking, and must avoid using wood and agricultural residues for cooking and should also refrain from tobacco smoking.

## Introduction

Asthma is a chronic inflammatory disease of airways of the lungs [1]. According to the Global Asthma Network report, Global Initiative for Asthma (GINA), approximately 339 million people, including six million children have bronchial asthma [2]. Globally, around three billion people and 90% of rural households in developing countries rely on kerosene, biomass and coal as primary source of cooking energy, typically burnt indoors in open fires often causing extreme pollution [3, 4] .

There are a lot of risk factors associated with the occurrence of asthma, such as indoor biomass cooking, outdoor pollution and occupational exposure, particularly in developing countries [5, 6]**.** Variations in home characteristics, household crowding, indoor smoke and poverty have been independently associated with asthma [7]**.** Researches show that biomass fuel is associated with increase in the risk of developing of asthma by increasing the concentration of indoor air pollutants [8, 9]. Not only biomass fuel, but also indoor cooking with gas stoves are positively associated with both allergy and asthma prevalence [10]. In contrast to the above studies, another research concludes that there is no significant association between indoor air pollutants from biomass fuel use and respiratory diseases, especially asthma [11]**.** The risk factors for asthma have been identified, but the cause of asthma is not known [12]. Social, psychological and physical factors in the environment may increase the risk for Asthma, including poverty, housing conditions and indoor allergen exposures [13]. There are conflicting evidences regarding the association between selected factors (indoor fuel exposure and housing characteristics) and asthma [10, 11, 14].

Most of the researches on implicated associations between asthma environmental factors are done in developed countries [10, 14]. In Africa, only few studies were conducted [8, 14]. Further, in Ethiopia, few studies tried to assess the risk factors for asthma [15]. It is inappropriate to assume that previously observed relationships between household fuel exposure, housing characteristics and chronic respiratory diseases apply in all contexts [9].

Despite the considerable population at risk worldwide, the effect of exposure to indoor solid fuel smoke has not been adequately studied [16]. There are studies that describe the relation between chronic respiratory diseases and their risk factors. Nevertheless, only few studies explore potential risk factors for asthma, particularly in Ethiopia [17]. Therefore, this study was intended to fill the information gap, particularly in Ethiopia, by studying the association between household fuel exposures and housing characteristics with asthma in Addis Ababa, Ethiopia.

## Methods

### Study design, area and period

A hospital based, matched case control study design was employed. Two referral hospitals, Tikur Anbessa Specialized Hospital (TASH) and St. Paul Hospital Millennium Medical College (SPHMMC) constituted study facilities. We used availability of spirometry for the diagnosis of asthma, case load (referrals from all over the country) and existence of specialized units (chest unit and internal medicine OPD) as criteria to select the hospitals. The study was conducted between February 1 and August 30, 2020 in Addis Ababa, Ethiopia.

### Study population

The study population comprised of all patients attending study hospitals (chest clinic and internal medicine OPD) in the designated study period. The eligibility criteria for Cases- were Spirometry confirmed asthmatic patients on follow up in the study hospitals chest clinic or internal medicine OPD and whose age were 18 years or older. Controls- were attendees of study hospitals in non-chest clinics of internal medicine OPD for non-asthma health problems. Matched with sex and age, two controls were selected for cases, in the same 5 year age group. (I.e. control age = case age ± 2 years). We excluded those cases and controls suffering from COPD, lung cancer, PTB and pneumonia and controls with clinical sign or symptom suggestive of asthma.

### Sample size calculation

Sample size was calculated using Epi info version 7 with the assumption of double population formula. The ratio of control to case is (R) 2:1. The prevalence of exposure (selected determinants, such as household biomass fuel exposure, household fossil fuel exposure and household crowding index) among controls are estimated from previous studies that are 10, 19.5 and 34% respectively [18–20]**.** Then, after adjusting for 5% non-response rate, the maximum sample size was taken corresponding to household fossil fuel exposure which were 487 (162 cases and 325 controls).

### Sampling procedure

From each hospital, participants were selected by consecutive/sequential sampling technique based on the proportion of selected hospitals total patient flow as denominator. Based on the proportional allocation total number of patients were 162, including 104 from TASH and 58 from SPHMMC. For each asthma cases, two age and sex matched controls were selected. Both cases and controls were selected from the same hospitals to control for the influence of context variation.

### Study variables

#### Dependent variable

Asthma status (cases and controls).

#### Independent variables

**Socio-demographic characteristics: (**Age, Residence, Sex, Marital status, Educational status, Occupational status, Monthly income, Religion).

**Housing characteristics:** (Number of rooms, Number of persons, Household crowding index, Lighting source, Cooking area, Cooking room floor material, Cooking room wall material, Cooking room roof material, Window in cooking room, Window opening, Door opening, Smoke extraction, Cooking time).

**Household fuel exposure characteristics:** (Stove type (Three stone fire, Shielded mud stove, Wood burning metal stove, Improved charcoal stove, Kerosene stove, LPG (gas) stove, Solar cooker, Grid powered electric stove), Current household fuel type (Wood, Charcoal, Kerosene, Dung, Agricultural residues, Solar, Grid electricity) and Life time household fuel type (Wood, Charcoal, Kerosene, Dung, Agricultural residues, Solar, Grid electricity)),

**Pet ownership: (**Pet ownership (cat, dog, bird) and Contact with them).

**Life style characteristics:** (Tobacco smoking status, Shisha smoking status, Alcohol drinking, Chat chewing status, Drug using status, Family history of asthma, Physical activity).

**Nutritional status:** (Body mass index, Weight, Height).

**Co-morbidities** (Cardiac, Hypertension, Diabetes, Nerve, Kidney disease, Liver disease, HIV).

### Variables and measurements

- **Asthma** – is defined based on respiratory symptom typical of asthma and spirometry result (reversibility of airway obstruction i.e. greater than 12% and 200 ml increase in FEV1 from baseline) following inhalation of bronchodilator [2] .
- **Biomass fuel user-** In the questionnaire, an individual was considered as biomass fuel user, if he/she used fuel, including wood, charcoal, plant residues or agricultural waste and animal dung, mainly from domesticated animals for cooking or heating purposes. Pertaining to user classification, former biomass user means previously used biomass fuel, but stopped or not used biomass fuel with in the last 12 months, current biomass user means that uses biomass fuel with in the last 12 months and if users either current, former or both called life time user [21, 22]**.**
- **Fossil fuel user -** In the questionnaire, an individual is considered as fossil fuel user if he/she, uses fuel such as oil, coal, natural gas, kerosene and liquid petroleum gas for cooking. Regarding user classification, former fossil fuel user means previously used fossil fuel, but stopped or not used fossil fuel with in the last 12 months, current fossil fuel user means that used fossil fuel with in the last 12 months and if users either current, former or both called life time user [21, 23] **.**
- **Household crowding index: -** denoted by the number of co-residents per room**.** i.e. number of co residents (excluding newborn) divided by number of rooms (excluding kitchen and bathroom) based on this it can be categorized in to three, low when it is less than one, medium when it is between one and two, and high when it is greater than three or more [24].
- **Window characteristics**- Cooking room shall have at least one window, the minimum total window size shall be at least 8 % of the floor area of the room and ask participants for opening during cooking [25].
- **Door characteristics;** the environment has an opening in the windward façade and a door in the leeward façade and the minimum requirement door characteristics are width of 0.80 m and height of 2,10 m and ask participants for opening during cooking [26] .
- **Cooking time:** cooking time in this study was measured by asking the following question “how many minutes on average do you spend preparing, cooking and cleaning up each time per day?” And the answer were recorded in to three categories, less than 60 min, between 60and 120 min, and greater than 120 min [27].

### Data collection procedure

Data were collected through face to face interview by trained data collectors. The data collection tool used in this study was adopted from the previously peer reviewed studies, European Community Respiratory Health Survey II (ECRHS II) questionnaires and American Thoracic Society division of lung disease (ATS-DLD-78) questionnaires by combining them in to one, and appropriate modifications were made to serve our purpose [28, 29]. The first part addressed demographic and socioeconomic characteristics, while the second part included items related to potentials risk factor predictors, including household cooking, housing characteristics, cooking room characteristics, smoking status, alcohol drinking, family history of asthma, pet ownership, physical activity and obesity. A screening questionnaire was used to exclude any one with asthmatic symptoms from control groups. The survey questionnaire was used for both cases and controls. Data regarding patient’s medical information were extracted from patient cards.

### Data quality management

Eight data collectors (Nurses) and two supervisors (Master in Public Health) participated. Training was given for 3 days on the content of questionnaire, data collection methods, interview techniques, ethical concerns and the purpose of the study. The questionnaire prepared in English was translated to Amharic and back to English to maintain consistency of the questions. Pre testing of the questionnaire was made in Yekatit 12 Hospital on 5% of study population to ensure the quality of data. Completeness and consistency of the questionnaire were assessed daily by supervisors. Data were entered in to a template prepared on Epi-Info software and inconsistencies on the entered data were reconciled by checking the questionnaire. Data cleaning was done by running frequencies and cross-tabulating with the main outcome variables. Before analysis, missing values and outliers were checked. The data were exported to SPSS version 24 for analysis.

### Data analysis procedure

First, descriptive statistics were computed using frequency distribution and proportions for categorical variables and, mean and standard deviations (SD) for continuous variables were described. Chi-square (x^2^) test was used to assess the level of significant differences. A threshold *p* value of less than < 0.05 was used to declare significant association between asthma and its predictors in the chi-square (x^2^) test. Since we used matched case control study design conditional logistic regression were performed. Hence, variables which were found to be associated with asthma were included in the conditional binary logistic regression. Furthermore, different variables were included in the conditional multivariable logistic regression to single out the effect of each covariate with asthma and adjusted odds ratios; with 95% confidence interval was considered to see the association. Confounders were checked to minimize bias. The model goodness of the test was checked by Hosmer - Lemeshow goodness of fit test. The model is fit at x^2^ = 3.711 and *p*-value of 0.882. A 95% confidence interval for crude and adjusted ORs and a threshold p-value of < 0.05 were used to decide significance of associations.

### Ethical consideration

Before conducting the study ethical clearance was secured from the Research Ethical Committee of the School of Public Health, College of Health Sciences of the Addis Ababa University. Data collectors got written informed consent from the participants after informed them clearly about the aim of the study and the fact that it has no invasive procedure and harm. Respondents were informed that they could refuse or discontinue participation at any time and they were informed of the fact that Information is recorded without their names being mentioned. Only codes were used to keep it anonymous and maintain confidentiality and privacy of respondents.

## Results

### Descriptive analysis

#### Socio-demographic characteristics of study participants

From those patients who came for different services at TASH and SPHMMC, out of 487 participants approached, four people were excluded (1 case and 3 controls) due to failure to get age and sex matched controls. A total of 483 patients were enrolled in the study, which makes the response rate 99.17%. Spirometry was performed for all 162 cases to confirm the diagnosis of asthma. Baseline spirometry with repeat testing after 15 min to demonstrate reversibility (increase in FEV1 of 12% and 200 ml) after the administration of bronchodilator (salbutamol) was performed. Based on this procedure, patients with an obstructive pattern and reversibility on spirometry were included as cases. Of the 162 subjects, FEV1 was < 60% in 125 (77.16%) and 60–80% in 37 (22.84%).

Among the participant of the study, majority of proportion of ages lies between the age group of 41–60, which shows that 79 (49.1%) cases and 160 (49.7%) respectively and the mean _±_SD age of participants was 49.8 ± 14.1 and 49.4 ± 13.8 for cases and controls respectively. From total participants, more cases 7(4.3%) than controls (7(2.2%) were from rural area. Most of participants were female 98(60.9%) of cases and 196(60.9%) of controls. Majority 97(60.2%) cases and 225(69.9%) controls were married. With respect to educational status, majority of participants 51(31.7%) of cases and 95 (29.5%) controls attained primary education and with regard to occupational status, majority of participants were employed either in government, private or self-87(54%) of cases and 193(59.9%) of controls. The average monthly income mean ± SD for cases and controls were 2790.0 ± 2923.8 and 2609.7 ± 2459.2 ETB respectively. Majority 104(64.6%) of cases and 218(67.7%) controls were orthodox (Table 1).

**Table 1:** Socio-demographic characteristics of participants in selected public hospitals of Addis Ababa, Ethiopia, 2020

Variables	Cases	Control	Chi-square (x^**2**^)	***P*** value
	Number (%)	Number (%)		
**Age:**				
≤20	0 (0)	2(0.6)		
21–40	45 (28)	93(28.9)	1.299	0.862
41–60	79 (49.1)	160(49.7)		
61–79	34 (21.1)	61(18.9)		
≥80	3 (1.9)	6(1.9)		
**Residence**:				
Rural	7(4.3)	7(2.2)	1.802	0.179
Urban	154(95.7)	315(97.8)		
**Sex:**				
Male	63(39.1)	126(39.1)	0.000	1
Female	98(60.9)	196(60.9)		
**Marital status:**				
Single	25(15.5)	45(14)	7.760	0.051
Married	97(60.2)	225(69.9)		
Widowed	30(18.6)	46(14.3)		
Separated/divorced	9(5.6)	6(1.9)		
**Educational status:**				
Unable to read and write	40 (24.8)	86 (26.7)		
Primary (1–8)	51 (31.7)	95 (29.5)	0.477	0.976
Secondary(9–10)	18 (11.2)	37 (11.5)		
Preparatory (11–12)	4 (2.5)	10 (3.1)		
Diploma and above	48 (29.8)	94 (29.2)		
**Occupational status:**				
Unemployed	55(34.2)	106(32.9)		
Employed	87(54)	193(59.9)	3.579	0.311
Retired	18(11.2)	21(6.5)		
Student	1(0.6)	2(0.6)		
**Monthly income:**				
Less than 500	41(25.5)	84(26.1)		
500–1500	35(21.7)	54(16.8)	5.236	0.155
1501–2500	17(10.6)	57(17.7)		
Greater than 2500	68(42.2)	127(39.4)		
**Religion:**				
Orthodox	104(64.6)	218(67.7)		
Muslim	19(11.8)	42(13)	6.474	0.091
Protestant	35(21.7)	46(14.3)		
Catholic	3(1.9)	16(5)		

Employed (govt,private,self), ETB (Ethiopian Birr)

#### Housing characteristics

Housing characteristics of cases and controls of the total study population were presented below in (Table 2), majority of person have 2 rooms 59(36.6%) of cases and 104(32.3%) of controls. On the other hand mean ± SD number of room was 2.4 ± 1.4 and 1.9 ± 0.9 for cases and controls, respectively. And also persons living in the house were four or greater than four which were represented by 102(63.4%) cases and 164(50.9%) controls and mean ± SD number of person was 4.4 ± 2.2 and 3.7 ± 1.8 for cases and controls, respectively. Among those participant household crowding index value lies between 1 and 2 were 70(43.5%) of cases and 151(46.9%) of controls and Household crowding index mean ± SD was 2.1 ± 1.1 of cases and 2.1 ± 0.8 of controls.. Majority of participant use electricity for lighting source 157(97.5%) of cases and 317(98.4%)of controls. A high proportion of cases and controls had enclosed area (kitchen) for cooking purpose reported among 117(72.7%) of cases and 241 (74.8%) of controls. When we try to see smoke extraction, majority of participant used door opening practice as ventilation system 76(47.2%) of cases and 162(50.3%) of controls. On the other hand majority of participants 74(46%) cases and 304(94.4) controls spent 60–120 min for cooking a day and mean ± SD of Cooking time in minute were represented by 97.1 ± 55.8 of cases and 94.7 ± 35.7 of controls respectively.

**Table 2:** Housing characteristics of participants in selected public hospitals of Addis Ababa, Ethiopia, 2020

Variables	Cases	Control	Chi-square (x^**2**^)	***P*** value
	Number (%)	Number (%)		
**Number of room:**				
1	44 (27.3)	86 (26.7)		
2	59 (36.6)	104 (32.3)	1.549	0.671
3	34 (21.1)	72 (22.4)		
4 or greater than 4	24 (14.9)	60 (18.6)		
**Number of person:**				
1	13(8.1)	44(13.7)		
2	15(9.3)	30(9.3)	7.954	0.047
3	31(19.3)	84(26.1)		
4 or greater than 4	102(63.4)	164(50.9)		
**Household crowding index:**				
Less than 1	31(19.3)	54(16.8)	0.676	0.713
1–2	70(43.5)	151(46.9)		
Greater than 2	60(37.3)	117(36.3)		
**Lighting source**				
Lanterns/gas	4(2.5)	5(1.6)	0.509	0.475
Electricity	157(97.5)	317(98.4)		
**Cooking area**				
Enclosed area (Kitchen)	117 (72.7)	241 (74.8)	0.265	0.876
Semi-open area	6 (3.7)	11 (3.4)		
Part of main living area	38 (23.6)	70 (21.7)		
**Smoke extraction/ ventilation**				
Permanent holes in roof	3(1.9)	0(0)		
Eaves spaces	1(0.6)	2 (0.6)	9.854	0.131
Chimney	5(3.1)	3 (0.9)		
Windows openings	4(2.5)	12 (3.7)		
Door opening	76(47.2)	162 (50.3)		
Door and window opening	70(43.5)	138 (42.9)		
Open area	2(1.2)	5 (1.6)		
**Cooking time**				
Less than 60 min	71(44.1)	2(0.6)	125.828	0.000
60–120 min	74(46)	304(94.4)		
Greater than 120 min	16(9.9)	16(5)		

#### Cooking room characteristics

Cooking room characteristics of cases and controls are presented. Majority of participant cooking room floor material were cement represented by 91(56.5%) of cases and 187(58.1%) of controls. With respect to cooking house wall material 63(39.1%) of cases and 94(29.2%) of controls, were iron sheet respectively. On the other hand most of participants cooking room roof material were iron sheet 154(95.7%) of cases and 304(94.4%) of controls. Based on participants report, 77(47.8%) of cases and 276(85.7%) of controls has windows in cooking room. Among those who haves it 76(47.2%) cases and 273(84.6%) controls practice window opening during cooking time. on the other hand 114(70.8%) cases and 299(92.9%) controls practice door opening during cooking time. The detail of cooking room characteristics of the study participants is presented in the following (Table 3).

**Table 3:** Cooking room characteristics of participants in selected public hospitals of Addis Ababa, Ethiopia, 2020

Variables	Cases	Control	Chi-square (x^**2**^)	***P*** value
	Number (%)	Number (%)		
**Cooking house floor material**				
Clay/mud	52 (32.3)	99 (30.7)	0.465	0.926
Cement	91 (56.5)	187 (58.1)		
ceramic	9 (5.6)	21 (6.5)		
wood	9 (5.6)	15 (4.7)		
**Cooking house wall material**				
Iron sheets	63(39.1)	94(29.2)	7.323	0.062
Burned bricks	38(23.6)	82(25.5)		
Wattle and daub	59(36.6)	146(45.3)		
Plastic	1(0.6)	0(0)		
**Cooking house roof material**				
Tiles	7 (4.3)	18 (5.6)	6.337	0.561
Iron sheets	154 (95.7)	304 (94.4)		
**Window in cooking house**				
No	84(52.2)	46(14.3)	78.328	0.000
Yes	77(47.8)	276(85.7)		
**Window opening**				
No	85(52.8)	49(15.2)	75.606	0.000
Yes	76(47.2)	273(84.6)		
**Door opening**				
No	47(29.2)	23(7.1)	42.110	0.000
Yes	114(70.8)	299(92.9)		

### Association of different characteristics of participant with asthma

The conditional multiple logistic regression analysis provided that people who didn’t practice door opening, while cooking were at high risk to develop asthma compared with people who had door opening practice while cooking. The odds of developing asthma was found to be 10 times higher among people who don’t have door opening practice compared to people who have door opening practice while cooking, (AOR:10.25, 95% CI, (3.97, 26.49)).

With regard to ever tobacco smoking status, the conditional bi variable analysis identified that, tobacco use as significant risk factor to develop asthma (COR: 8.23 (3.10, 21.84)). Its association also persisted when adjusted for confounders. As we hold all other factors in the model constant, the odds of developing asthma was found to be 6 times more likely among smoker compared to non-smokers, (AOR:6.16, 95% CI, (1.30, 29.10)).

Compared to people who hadn’t family history of asthma, people who had family history of asthma were 5 times more likely to develop asthma, after keeping all independent variables constant, (AOR:4.72, 95% CI, (1.54, 14.45)).

Participants who were life time wood user were at high risk to develop asthma. The conditional multivariable analysis indicated that people who use wood in their life time for cooking were 4 times at risk of developing asthma compared to people who didn’t use, (AOR:4.95, 95% CI, (2.1, 11.69)).

With respect to agricultural residues using status, people who use agricultural residues for cooking throughout their life time were found to be at risk to develop asthma compared to those who didn’t use agricultural residues for cooking in their life time. The odds of developing asthma was found to be 3 times higher among people who use agricultural residues for cooking compared to people who didn’t use agricultural residues throughout their life time for cooking, (AOR:3.81, 95% CI, (1.05, 13.79)). (Table 4).

**Table 4:** Conditional multivariable logistic regression analysis of factors associated with asthma among participants in selected public hospitals of Addis Ababa, Ethiopia, 2020

Variables	Cases	Control	COR at 95%, CI	AOR 95%, CI
	Number (%)	Number (%)		
**Number of person:**				
1	13(8.1)	44(13.7)	1	1
2	15(9.3)	30(9.3)	2.08 (0.83, 5.23)	4.10 (0.71, 23.76)
3	31(19.3)	84(26.1)	1.39 (0.63, 3.08)	2.54 (0.60, 10.82)
4 or greater than 4	102(63.4)	164(50.9)	2.52 (1.20, 5.31)	1.42 (0.30, 6.81)
**Window in cooking house**				
No	84(52.2)	46(14.3)	6.73 (4.10, 11.05)	1.92 (0.01, 47.77)
Yes	77(47.8)	276(85.7)	1	1
**Window opening**				
No	85(52.8)	49(15.2)	6.42 (3.95,10.43)	2.17 (0.01, 52.66)
Yes	76(47.2)	273(84.6)	1	1
**Door opening**				
No	47(29.2)	23(7.1)	5.13 (2.93, 8.96)	10.25 (3.97, 26.49)*
Yes	114(70.8)	299(92.9)	1	1
**Cooking time**				
Less than 60 mint	71(44.1)	2(0.6)	1	1
60–120 mint	74(46.0)	304(94.4)	1.03 (0.66, 1.61)	0.69(0.30, 1.59)
Greater than 120 mint	16(9.9)	16(5)	2.31 (1.05, 5.10)	1.61 (0.31, 8.23)
**Stove type:**				
**Three-stone fire**				
No	142(88.2)	316(98.1)	1	1
Yes	19(11.8)	6(1.9)	5.61 (2.36,13.37)	0.20 (0.01, 2.92)
**Kerosene stove**				
No	108(67.1)	95(29.5)	1	1
Yes	53(32.9)	227(70.5)	0.21 (0.14, 0.33)	0.20 (0.03, 1.34)
**Current household fuel:**				
**Wood**				
No	119(73.9)	312(96.9)	1	1
Yes	42(26.1)	10(3.1)	8.41 (4.21, 16.77)	5.48 (0.43, 69.52)
**Kerosene (Paraffin)**				
No Yes	55(34.2) 106(65.8)	102(31.7) 220(68.3)	1 0.23 (0.15, 0.35)	1 3.39 (0.45, 25.35)
**Dung**				
No	143(88.8)	315(97.8)	1	1
Yes	18(11.2)	7(2.2)	5.77 (2.28, 14.58)	0.62 (0.06, 6.46)
**Agricultural residues**				
No	145(90.1)	317(98.4)	1	1
Yes	16(9.9)	5(1.6)	7.65 (2.55, 22.96)	0.59 (0.04, 8.51)
**Lifetime household fuel:**				
**Wood**				
No	48(29.8)	239(74.2)	1	1
Yes	113(70.2)	83(25.8)	7.11 (4.41, 11.48)	4.95 (2.1, 11.69)*
**Charcoal**				
No	11(6.8)	59(18.3)	1	1
Yes	150(93.2)	263(81.7)	3.45 (1.73, 6.88)	1.36 (0.43, 4.33)
**Kerosene (Paraffin**)				
No	86(53.4)	53(16.5)	1	1
Yes	75(46.6)	269(83.5)	0.17 (0.10, 0.27)	0.61 (0.22, 1.69)
**Dung**				
No	107(66.5)	300(93.2)	1	1
Yes	54(33.5)	22(6.8)	7.89 (4.20, 14.84)	1.23 (0.31, 4.94)
**Agricultural residues**				
No	103(64)	299(92.9)	1	1
Yes	58(36)	23(7.1)	7.38 (4.1, 13.29)	3.81 (1.05, 13.79)*
**Pet**				
No	84(52.2)	249(77.3)	1	1
Yes	77(47.8)	73(22.7)	3.19 (2.09, 4.87)	1.97 (0.86, 4.49)
**Ever tobacco smoking status**				
No	139(86.3)	315(97.8)	1	1
Yes	22(13.7)	7(2.2)	8.23 (3.11, 21.84)	6.16 (1.30, 29.10)*
**Family asthma history**				
No	133(82.6)	309(96.0)	1	1
Yes	28(17.4)	13(4.0)	4.66 (2.36, 9.18)	4.72 (1.54, 14.45)*

* Significant at *P* < 0.05, 1 = reference, *COR* crude odds ratio, *AOR* adjusted odds ratio

## Discussions

We found that door opening practice while cooking as a protective factor for asthma. On the other hand, life time uses of wood and agricultural residues for cooking purpose and tobacco smoking were found to be significant risk factors for asthma. Family history of asthma was also noted to be another risk factor for asthma.

The odds of developing asthma was 10 times higher among individuals who did not practice door opening while cooking compared with individuals who practiced door opening during cooking (AOR: 10.25, 95% CI:3.97, 26.49). The association may be due to overcrowding and inadequate ventilation for the presence of smoke measured by elevated CO2 level, high level of benzene and other VOCs [7]. This may be responsible for the development of asthma. The finding on this study supported by different studies such as that of the study which was conducted in Alaska [7], in Japan [30] and Southern New England [31]. In contrast to that, another study conducted in Peru provides unequivocal report which state that there is no association between door opening practice and asthma [32]. This variation may occur due to area of cooking room and other smoke extraction system.

Those subjects who used agricultural residues in their life time for cooking were almost four times more likely to develop asthma compared to people, who don’t use agricultural residues in their life time for cooking (AOR: 3.81, 95% CI: 1.05, 13.79). Similarly, the use of wood in their life time for cooking purpose was found to be important predictor for acquiring of asthma in this study. The odds of developing asthma was almost five times higher among subjects who use wood for cooking compared to subjects who do not use wood (AOR: 4.95, 95% CI: 2.1, 11.69). Finding from this study suggest that the risk of wood smoke exposure for asthma may be confined due to their duration of exposure to the smoke and compromised respiratory system from cooking smoke.

The association between biomass fuel smoke exposure and asthma are confirmed by different researches for instance studies conducted in Turkey [33], in Nigeria [34] and in India [9, 35]. The assumption for this may be smoke emanating from burning biomass fuels contains toxic pollutants called oxidants, which include volatile organic compound (VOCs), particulate matter (PM), carbon monoxide (CO), and oxides of nitrogen, sulfur and Florine [36]. Because of this oxidative stress, which is imbalance between biological pro-oxidants and anti-oxidant defense system, some changes observed like initiate cytokine production and depression of protective membrane and mediates inflammatory response or worsen asthma due to a compromised anti-oxidant defense system [36]. On the other hand, study conducted in Peru [6] and in Nigeria [37] have showed lack of association between biomass fuel smoke exposure and asthma. The discrepancies between studies may be possible due to variations in cooking area, ventilation system, as well as in type, duration and intensity of biomass fuel smoke exposure.

Tobacco smoking has also been identified as important risk factor for developing asthma by six times (AOR: 6.16, 95% CI: 1.3, 29.1). Our study yield similar finding with the study conducted in Thailand which state that there is a significant association between smoking and asthma [38]. The justification for this may be the effect of smoking on airway i.e. tobacco smoke flow inward to inflammatory cell like neutrophils, lymphocytes, eosinophils, mast cell and macrophages [39]. Since various inflammatory mediators are released which include lipids, chemokines, cytokines and growth factor and those proteolytic enzymes cause inflammatory damage and bronchial hyper-responsiveness which is hall mark of asthma [39]. On the other hand studies conducted in Britain [40] and Uganda [41] are in contrast with our finding stating that there is no significant association between tobacco smoking and asthma. This could be due to variation in the study population, study design and magnitude of cigarette smoking.

Patients who had family history of asthma were more than four times more likely to develop asthma compared to those who had no family history of asthma (AOR: 4.72, 95% CI: 1.54, 14.45). The possible explanation for this association could be either due to hereditary factors or a shared environment by the member of a family contributes to the pathogenesis of asthma [6]. The finding of our study is supported by other studies which were conducted in Australia [38], Thailand [42], Uganda [43], New Zealand [44] and California [45], which concluded that family history of asthma is one of the major risk factor for the development of asthma. The justification for the similarity might be the study setting in which most of the studies were conducted in a hospital setting. However, another study conducted in Britain is not in agreement with our finding, which states that there is no significant relationship between family history of asthma and asthma (42). This may be due to study design and population characteristics.

This study has some limitations. First, assessment of housing characteristics information was obtained by interview, but it would be better if we obtain it through direct physical observation. Since the study was done in two referral hospitals, it may also be affected by selection bias or it might not be generalized to the population with asthma despite our effort to incorporate all referral hospitals which used spirometry to increase its representation. Finally based on our study design and subjects, it is difficult to identify the exact causes of asthma, and we recommend that a further prospective large scale study should be done to identify causes of asthma.

## Conclusions

Use of Wood and agricultural residues for cooking purpose in life time were found to be significant risk factors for asthma. Tobacco smoking was also considered to be another risk for asthma. On the other hand, opening door while cooking was founds to be preventive for asthma. Family history of asthma was noted to have its own contribution for the development of asthma. To reduce the risk of asthma, people should practice door opening while cooking, and must limit and avoid using wood and agricultural residues for cooking and refrain from tobacco smoking.

## Data Availability

The datasets used and/or analysed during the current study are available from the corresponding author on reasonable request.

## Abbreviation

ATS: American Thoracic Society
BMI: Body Mass Index
COPD: Chronic Obstructive Pulmonary Disease
CI: Confidence Interval
COR: Crude Odds Ratio
CRD: Chronic Respiratory Diseases
DALYS: Disability-adjusted life years
ECRHS II: European Community Respiratory Health Survey II
GBD: Global Burden of Disease
GINA: Global Initiative for Asthma
IAQ: Indoor Air Quality
LRTI: Lower Respiratory Tract Infectious
MMMF: Man Made Mineral Fibers
MOR: Matched Odd Ratio
NCDs: Non Communicable Diseases
OPD: Outpatient Department
OR: Odds Ratio
PTB: Pulmonary Tuberculosis
SD: Standard Deviations
SPHMMC: St, Paul Hospital millennium medical college
SPSS: Statistical Package for Social Sciences
TASH: Tikur Anbessa Specialized Hospital
VOC: Volatile Organic Compound
WHO: World Health Organization
